# Supplementary material for: p53 promotes revival stem cells in the regenerating intestine after severe radiation injury
Source: Nat Commun. 2024 Apr 8;15:3018. doi: 10.1038/s41467-024-47124-8 (PMC11001929; doi:10.1038/s41467-024-47124-8)
Supplement: Supplementary file 3 — Description of Additional Supplementary Files [file 41467_2024_47124_MOESM3_ESM.pdf]

Description of Additional Supplementary Files

File Name: Supplementary Data 1
Description: Differentially expressed genes in each cell cluster.

File Name: Supplementary Data 2
Description: Publicly available gene signatures used in this study.
