# Supplementary material for: p53 promotes revival stem cells in the regenerating intestine after severe radiation injury
Source: Nat Commun. 2024 Apr 8;15:3018. doi: 10.1038/s41467-024-47124-8 (PMC11001929; doi:10.1038/s41467-024-47124-8)
Supplement: Supplementary file 6 — Reporting Summary [file 41467_2024_47124_MOESM6_ESM.pdf]

nature portfolio

Corresponding author(s): Jeffrey L. Wrana,C hang-Lung Lee,D avid G. Kirsch

Last updated by author(s): Mar 3, 2024

Reporting Summary

Nature Portfolio wishes to improve the reproducibility of the work that we publish. This form provides structure for consistency and transparency in reporting. For further information on Nature Portfolio policies, see our Editorial Policies and the Editorial Policy Checklist.

Statistics

For all statistical analyses, confirm that the following items are present in the figure legend, table legend, main text, or Methods section.

n/a	Confirmed	
☐	☒	The exact sample size (*n*) for each experimental group/condition, given as a discrete number and unit of measurement
☐	☒	A statement on whether measurements were taken from distinct samples or whether the same sample was measured repeatedly
☐	☒	The statistical test(s) used AND whether they are one- or two-sided<br>*Only common tests should be described solely by name; describe more complex techniques in the Methods section.*
☒	☐	A description of all covariates tested
☐	☒	A description of any assumptions or corrections, such as tests of normality and adjustment for multiple comparisons
☐	☒	A full description of the statistical parameters including central tendency (e.g. means) or other basic estimates (e.g. regression coefficient) AND variation (e.g. standard deviation) or associated estimates of uncertainty (e.g. confidence intervals)
☐	☒	For null hypothesis testing, the test statistic (e.g. *F*, *t*, *r*) with confidence intervals, effect sizes, degrees of freedom and *P* value noted<br>*Give P values as exact values whenever suitable.*
☒	☐	For Bayesian analysis, information on the choice of priors and Markov chain Monte Carlo settings
☐	☒	For hierarchical and complex designs, identification of the appropriate level for tests and full reporting of outcomes
☒	☐	Estimates of effect sizes (e.g. Cohen's *d*, Pearson's *r*), indicating how they were calculated

Our web collection on statistics for biologists contains articles on many of the points above.

Software and code

Policy information about availability of computer code

Data collection	- RNA extraction and preparation of the gene expression libraries for single-cell RNA sequencing the Chromium Next GEM Single Cell 3' v3.l Kit from l0X Genomics was used.<br>- Gene expression libraries were sequenced using an Illumina NovaSeq 6000 platform.<br>- IHQ images of intestinal tissue sections were acquired using a Leica DM2000 LED phase contrast microscope.<br>- IF and ISH images of intestinal tissue sections and intestinal organoids were acquired using a Nikon Ti2 inverted confocal microscope system.<br>- For survival analysis and in vivo lineage tracing experiments subtotal body irradiation was performed using a an X-RAD 320 biological irradiator .<br>- For single-cell RNA sequencing subtotal body irradiation was performed using a 3rd generation Small Animal Radiation Research Platform (SARRP by XStrahl).<br>- Intestinal Organoids were irradiated using a GammaCell 40 irradiator.<br>- BD Influx System (serial number X5000032) was used to sort DAPI negative cells from harvested intestinal tissues to obtain single and viable cells for downstream single cell RNA sequencing.<br>-Fluorescence in serum samples was measured using an Infinite Pro2000 plate reader.
Data analysis	- Cell Ranger pipeline from lOX genomics was used to demultiplex FASTQ reads, align them to the mm1O Mouse genome, and generate gene-barcode expression matrices.<br>- R 4.2.1 was used to analyze single cell RNA sequencing data. Several R packages were used for the analysis including: Seurat v4.3.0, ensembldb v2.22.0, ggplot2 v3.4.0, AnnotationHub v3.6.0, ClusterProfiler v4.6.0, phetmap v1.0.12, RColorBrewer v1.1-3, msigdbr v7.5.1, harmony v1.2.0

- Cell Profiler was used to quantify the number of p53 positive cells in IF intestinal tissue sections.
- Graphpad Prism 9 and R v4.3.1 were used for generating plots and for statistical analysis.
- QuPath v3.3.0 was used to quantify the number of Clu, Mki67 and Ly6a positive cells in intestinal tissue sections.

For manuscripts utilizing custom algorithms or software that are central to the research but not yet described in published literature, software must be made available to editors and reviewers. We strongly encourage code deposition in a community repository (e.g. GitHub). See the Nature Portfolio guidelines for submitting code & software for further information.

Data

Policy information about availability of data

All manuscripts must include a data availability statement. This statement should provide the following information, where applicable:

- Accession codes, unique identifiers, or web links for publicly available datasets
- A description of any restrictions on data availability
- For clinical datasets or third party data, please ensure that the statement adheres to our policy

The single-cell RNA sequencing data generated in this study have been deposited in the SRA database under accession code SRR22589001 [https://www.ncbi.nlm.nih.gov/sra/?term=SRR22589001]. The single-cell RNA sequencing data from Ayyaz et al. Nature 2019 and the single-cell RNA sequencing data used in Supplementary Figure 2 are available from Gene Expression Omnibus (GEO) with accession codes GSE123516 and GSE152376, respectively.

Research involving human participants, their data, or biological material

Policy information about studies with human participants or human data. See also policy information about sex, gender (identity/presentation), and sexual orientation and race, ethnicity and racism.

Reporting on sex and gender

Use the terms sex (biological attribute) and gender (shaped by social and cultural circumstances) carefully in order to avoid confusing both terms. Indicate if findings apply to only one sex or gender; describe whether sex and gender were considered in study design; whether sex and/or gender was determined based on self-reporting or assigned and methods used.
Provide in the source data disaggregated sex and gender data, where this information has been collected, and if consent has been obtained for sharing of individual-level data; provide overall numbers in this Reporting Summary. Please state if this information has not been collected.
Report sex- and gender-based analyses where performed, justify reasons for lack of sex- and gender-based analysis.

Reporting on race, ethnicity, or other socially relevant groupings

Please specify the socially constructed or socially relevant categorization variable(s) used in your manuscript and explain why they were used. Please note that such variables should not be used as proxies for other socially constructed/relevant variables (for example, race or ethnicity should not be used as a proxy for socioeconomic status).
Provide clear definitions of the relevant terms used, how they were provided (by the participants/respondents, the researchers, or third parties), and the method(s) used to classify people into the different categories (e.g. self-report, census or administrative data, social media data, etc.)
Please provide details about how you controlled for confounding variables in your analyses.

Population characteristics

Describe the covariate-relevant population characteristics of the human research participants (e.g. age, genotypic information, past and current diagnosis and treatment categories). If you filled out the behavioural & social sciences study design questions and have nothing to add here, write "See above."

Recruitment

Describe how participants were recruited. Outline any potential self-selection bias or other biases that may be present and how these are likely to impact results.

Ethics oversight

Identify the organization(s) that approved the study protocol.

Note that full information on the approval of the study protocol must also be provided in the manuscript.

Field-specific reporting

Please select the one below that is the best fit for your research. If you are not sure, read the appropriate sections before making your selection.

[x] Life sciences [ ] Behavioural & social sciences [ ] Ecological, evolutionary & environmental sciences

For a reference copy of the document with all sections, see nature.com/documents/nr-reporting-summary-flat.pdf

Life sciences study design

All studies must disclose on these points even when the disclosure is negative.

Sample size

No statistical methods were used to determin the sample size. Sample size is indicated in figure legends of the manuscript. In brief, for the single cell RNA sequencing at least 2 mice/condition were processed and pooled together. For survival experiments between 5 and 20 mice were analyzed. For ISH analysis of Clu, Mki67 and Ly6a 3 mice for each condition were used and between 4-20 different areas of each small intestine were quantified. For IHQ quantification ofp21, p53 and CC3 at least 3 mice/condition were analyzed.

Data exclusions	For the single cell RNA sequencing cells that did not pass the quality control (see material and methods) were excluded from the downstream analysis. No other data was excluded from this study.
Replication	The in vitro experiments with intestinal organoids were replicated independently at least three times to confim reproducibility and statistical significant differences. Number of replicates are indicated in the figure legends.
Randomization	Mice were allocated randomly to different groups for irradiation treatments.
Blinding	All images were quantified by blinded observers to the treatment.

Reporting for specific materials, systems and methods

We require information from authors about some types of materials, experimental systems and methods used in many studies. Here, indicate whether each material, system or method listed is relevant to your study. If you are not sure if a list item applies to your research, read the appropriate section before selecting a response.

Materials & experimental systems

n/a	Involved in the study	
☐	☒	Antibodies
☐	☒	Eukaryotic cell lines
☒	☐	Palaeontology and archaeology
☐	☒	Animals and other organisms
☒	☐	Clinical data
☒	☐	Dual use research of concern
☒	☐	Plants

Methods

n/a	Involved in the study	
☒	☐	ChIP-seq
☐	☒	Flow cytometry
☒	☐	MRI-based neuroimaging

Antibodies

Antibodies used

Antibodies used in IHQ staining:
- Anti-p21 (Ref: ab188224; Clone: EPR18021; Supplier: Abcam)
- Anti-Cleaved caspase-3 (Asp175) (Ref: #9664; Clone:5A1E; Supplier: Cell Signaling Technology)
- Anti-p53 (Ref: NCL-L-p53-CM5p; Supplier: Leica Biosystems)

For chromogenic detection:
- Goat anti-Rabbit lgG (H+L) Cross-Adsorbed Secondary Antibody, Biotin (Ref:#31822; Supplier: lnvitrogen)

Antibodies used in IF staining:
- Anti-p53 (Ref: NCL-L-p53-CM5p; Supplier: Leica Biosystems).
- EpCAM (CD326) (Ref: 118201 ; Clone: G8.8; Supplier: Biolegend)
- GFP (Ref: #ab13970; Supplier: Abcam)

Secondary Dylight antibodies (Fisher Scientific).

The ISH probes to detect Ly6a, Clu, MKi67 and Mdm2 mRNA :
- Mmly6a-427571 (Supplier: ACDBio)
- MmCLuC3-427891-C3 (Supplier: ACDBio)
- Mmki67C2-416771 (Supplier: ACDBio)
- MmMdm2C2-447641-C2 (Supplier: ACDBio)

The Opal Fluorophore reagent packs:
Opal 520 (Ref: FP1487001KT; Supplier: Akoya Biosciences)
Opal 570 (Ref: FP1488001KT; Supplier: Akoya Biosciences)
Opal 690 (Ref: FP1497001KT; Supplier: Akoya Biosciences)

Tyramide signal amplification (TSA) fluorophores:
TSA Fluorescein, TSA Cyanine 3, TSA Cyanine 5 (Supplier: Akoya Biosciences)

Validation

The antibodies used in this studiy are all comercial available:

l. p21 (https://www.abcam.com/products/primary-anti bodies/p21-a ntibody-epr18021-ab188224.html)
2. cleaved caspase-3 (https://www.cellsignal.com/products/primary-antibodies/cleaved-caspase-3-asp 175-anti body /9661?Ns=product.currentlot.numberOfApplications%7Cl&N=2385223652+4294967292+4294956287&Nrpp=l00&No=%7Boffset%7D&fromPage=plp&bvstate=pg:2/ct:r&gclid=CjwKCAjw_MqgBhAGEiwAnYOAenYRZhb0IKKtSipGqyWG-mz7MtmEZtclzH2QULSyQhEHbx2qUBxuXhoCtUEQAvD_BwE&gclsrc=aw.ds)

3. p53 (https://shop. leica biosystems.com/us/i hc-ish/ihc-primary-antibodies/pid-p53-protein-cm5)
4. Epcam (https ://www. biol egen d .com/fr-ch/prod ucts/p u rifled-a nti-mou se-cd32 6-ep-ca m-a nti body-4 72 4)
5. GFP (https://www.abcam.com/products/primary-antibodies/gfp-antibody-ab13970.html)
6. Goat anti-Ra bit (https ://www. th ermofi sher. com/ anti body/product/Goat -anti-Rab bit -lgG-H-L-Cross-Ad so rbe d-Secon da ry-Anti body-Polyclona1/31822)
7. Secondary ab: Alexa Fluor 488 AffiniPure Goat AntiChicken lgY lgG HL #103-545-155 (cedarlane)
Donkey anti-rabbit lgG (H+L) Dylight 650 #SA5-10041(invitrogen)
8. Opal 520 ,570,690 reagent pack (https://www.akoyabio.com/phenoimager/assays/opal-fluorophore-reagent-packs/)
9. TSA Fl uorescein (https://www.akoyabio.com/phenoimager /assays/tsa-fl uorescein/)
10. TSA Cyanine 3 (https://www.akoyabio.com/phenoimager/assays/tsa-cyanine-3/)
11. TSA Cyanine 5 (https://www.akoyabio.com/phenoimager/assays/tsa-cyanine-5/)

Eukaryotic cell lines

Policy information about cell lines and Sex and Gender in Research

Cell line source(s)

The cell lines used in this study were small intestinal-derived organoids from genetically engineered mouse models. The mice strains used to derive the organoids are reported in the material and methods of the manuscript.

Authentication

Genotyping was performed on the mice used to derive the organoids

Mycoplasma contamination

The organoids were tested negative for mycoplasma contamination.

Commonly misidentified lines
(See ICLAC register)

No commonly misidentified cell lines were used in this study.

Animals and other research organisms

Policy information about studies involving animals; ARRIVE guidelines recommended for reporting animal research, and Sex and Gender in Research

Laboratory animals

The laboratory animals used in this study were commercially available, obtained from other laboratories or in-house bread. All animals used in this study had between 8-10 weeks of age. Animals were

Villin-Cre (Obtained from Jackson Laboratory)
p53FL/FL (Provided by A. Berns at the Netherlands Cancer Institute, Amsterdam, the Netherlands).
p53 FL/- (Created and bred at Duke University) p53LSL-25,26 (Provided by L. Attardi, Stanford University, Stanford, CA)
p53 LSL-25,26,53,54 (Provided by L. Attardi, Stanford University, Stanford, CA)
Clu-EGFP (Rockefeller University,GENSAT project)
Clu-CreERT2 (Created and bred at the Lunenfeld-Tanenbaum Research Institute)
Lgr5-CreERT2 (Obtained from Jackson Laboratory)
LgrS-GFP-iDTR (Provided by Frederic de Sauvage, Genentech, San Francisco, CA)
Ai9 and Ai14 (Obtained from Jackson Laboratory)

Wild animals

No wild animals were used in this study.

Reporting on sex

Males and female mice were used in this study.

Field-collected samples

This study does not involve samples collected from the field.

Ethics oversight

All procedures with mice were approved by the Institutional Animal Care and Use Committee (IACUC) of Duke University, by the IACUC of the University of Pennsylvania and by the Canadian Council on Animal Care.

Note that full information on the approval of the study protocol must also be provided in the manuscript.

Plants

Seed stocks

Report on the source of all seed stocks or other plant material used. If applicable, state the seed stock centre and catalogue number. If plant specimens were collected from the field, describe the collection location, date and sampling procedures.

Novel plant genotypes

Describe the methods by which all novel plant genotypes were produced. This includes those generated by transgenic approaches, gene editing, chemical/radiation-based mutagenesis and hybridization. For transgenic lines, describe the transformation method, the number of independent lines analyzed and the generation upon which experiments were performed. For gene-edited lines, describe the editor used, the endogenous sequence targeted for editing, the targeting guide RNA sequence (if applicable) and how the editor was applied.

Authentication

Describe any authentication procedures for each seed stock used or novel genotype generated. Describe any experiments used to assess the effect of a mutation and, where applicable, how potential secondary effects (e.g. second site T-DNA insertions, mosiacism, off-target gene editing) were examined.

Flow Cytometry

Plots

Confirm that:

- [x] The axis labels state the marker and fluorochrome used (e.g. CD4-FITC).
- [x] The axis scales are clearly visible. Include numbers along axes only for bottom left plot of group (a 'group' is an analysis of identical markers).
- [x] All plots are contour plots with outliers or pseudocolor plots.
- [ ] A numerical value for number of cells or percentage (with statistics) is provided.

Methodology

Sample preparation	Intestinal tissues from control or irradiated mice were harvested as desribed in the matherial and methods section. Single cells were resuspended in FACS buffer containing Adv-DMEM F12 and 0.lug/ml DAPI.
Instrument	BD Influx System (serial number X5000032)
Software	BD FACS'M Sortware vl.2.0.142
Cell population abundance	For the single cell RNA experiment we only sorted DAPI negative cells (alive). Cell viability was confirmed after sorting by using trypan blue and an automated cell counter (Countess 3, Invitrogen). All samples that were used for single cell RNA sequencing had more than 80% viability.
Gating strategy	DAPI negative cells were first selected using the 425/26[405] channel. From alive cells (DAPI negative) SSC vs FSC gatting was used to select all cells. From all alive cells, Trigger plus width vs FSC was used to select for single cells. We have provided an example for the gating strategy along with the sort reports in the Source Data file.

- [ ] Tick this box to confirm that a figure exemplifying the gating strategy is provided in the Supplementary Information.
