# Supplementary material for: p53 promotes revival stem cells in the regenerating intestine after severe radiation injury
Source: Nat Commun. 2024 Apr 8;15:3018. doi: 10.1038/s41467-024-47124-8 (PMC11001929; doi:10.1038/s41467-024-47124-8)
Supplement: Supplementary file 7 — Source data [file 41467_2024_47124_MOESM7_ESM.zip › Source_Data_Files/Example_Flow_Gating_Sorting.pdf]

Flow Gates Example: Sorting Intestinal Cells for sc-RNA sequencing

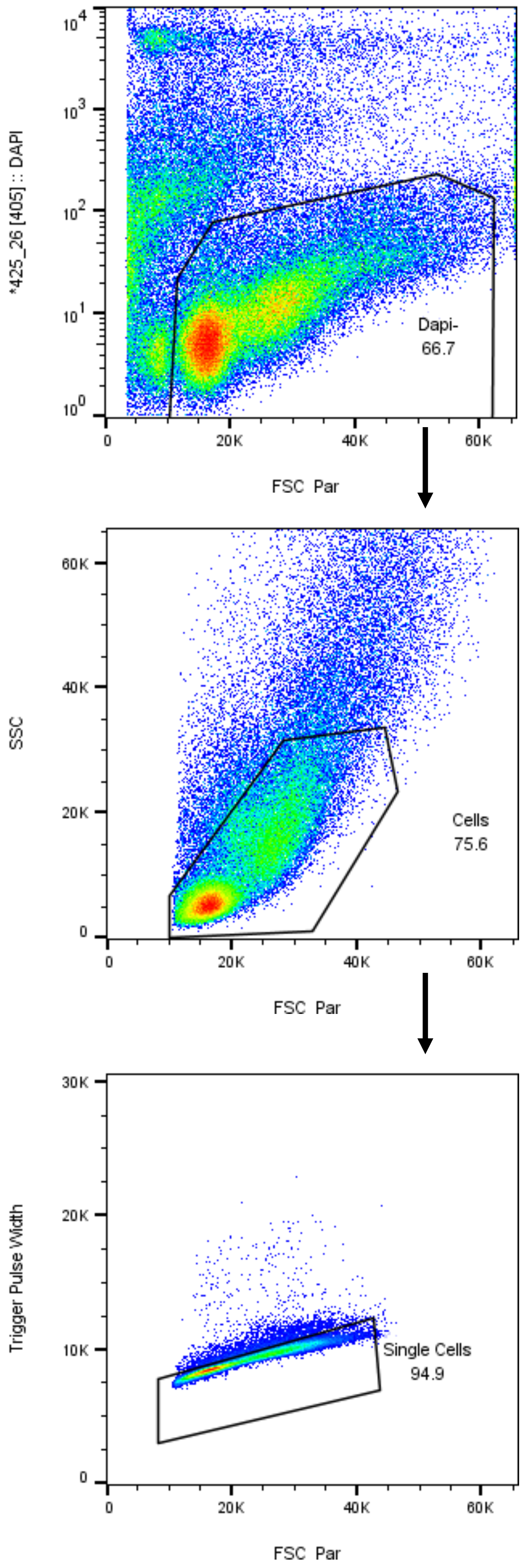

Sort Summary:

Sample 1_Rep1:

Sort Report

System

Sort Start:	12/1/2021 2:10:53 PM	Server:	Utopex
Application:	BD FACS™ Sortware	Build:	1.2.0.108
Version:	1.2.0.142	Cytometer Model:	BD Influx System (USB)
ValComp:	7.5.1.3.16	Cytometer Serial #:	X5000032

Details

Data Source:	Cytometer	Sort Mode:	1.0 Drop Pure
Nozzle Diameter (µm):	0.00	Drop Envelope:	1.0 Drop
Sheath Pressure (PSI):	0.00	Sort Objective:	Purify
Sort Device:	4 Tube Holder - 4 Way Sort	Phase Mask:	16/16
Piezo Amplitude:	6.00	Extra Coincidence Bits:	4
Drop Delay:	37.8125	Drop Frequency (kHz):	36.80

Sort Details

Name	Population	Event Limit	Event Count	Sort Count	Sort Rate	Aborts	Abort Rate	Efficiency	Time (sec)
Far Left	-	-	-	-	-	-	-	-	-
Left	P3	Unlimited	326,179	152,196	1,511	6,471	64	95.9%	100
Right	-	-	-	-	-	-	-	-	-
Far Right	-	-	-	-	-	-	-	-	-

Sample 1_Rep2:

Sort Report

System

Sort Start:	12/16/2021 2:14:44 PM	Server:	Utopex
Application:	BD FACS™ Sortware	Build:	1.2.0.108
Version:	1.2.0.142	Cytometer Model:	BD Influx System (USB)
ValComp:	7.5.1.3.16	Cytometer Serial #:	X64650000106

Details

Data Source:	Cytometer	Sort Mode:	1.0 Drop Pure
Nozzle Diameter (µm):	0.00	Drop Envelope:	1.0 Drop
Sheath Pressure (PSI):	0.00	Sort Objective:	Purify
Sort Device:	4 Tube Holder - 4 Way Sort	Phase Mask:	16/16
Piezo Amplitude:	7.35	Extra Coincidence Bits:	4
Drop Delay:	31.9000	Drop Frequency (kHz):	36.90

Sort Details

Name	Population	Event Limit	Event Count	Sort Count	Sort Rate	Aborts	Abort Rate	Efficiency	Time (sec)
Far Left	-	-	-	-	-	-	-	-	-
Left	P2	120,000	1,245,062	120,000	264	6,601	14	94.8%	453
Right	-	-	-	-	-	-	-	-	-
Far Right	-	-	-	-	-	-	-	-	-

Sample 2 _Rep1 :

Sort Report

System

Sort Start:	12/1/2021 2:06:37 PM	Server:	Utopex
Application:	BD FACS™ Sortware	Build:	1.2.0.108
Version:	1.2.0.142	Cytometer Model:	BD Influx System (USB)
ValComp:	7.5.1.3.16	Cytometer Serial #:	X5000032

Details

Data Source:	Cytometer	Sort Mode:	1.0 Drop Pure
Nozzle Diameter (µm):	0.00	Drop Envelope:	1.0 Drop
Sheath Pressure (PSI):	0.00	Sort Objective:	Purify
Sort Device:	4 Tube Holder - 4 Way Sort	Phase Mask:	16/16
Piezo Amplitude:	6.03	Extra Coincidence Bits:	4
Drop Delay:	37.8125	Drop Frequency (kHz):	36.80

Sort Details

Name	Population	Event Limit	Event Count	Sort Count	Sort Rate	Aborts	Abort Rate	Efficiency	Time (sec)
Far Left	-	-	-	-	-	-	-	-	-
Left	P3	Unlimited	402,434	152,181	906	5,300	31	96.6%	167
Right	-	-	-	-	-	-	-	-	-
Far Right	-	-	-	-	-	-	-	-	-

Sample 2 _Rep2 :

Sort Report

System

Sort Start:	12/16/2021 2:11:27 PM	Server:	Utopex
Application:	BD FACS™ Sortware	Build:	1.2.0.108
Version:	1.2.0.142	Cytometer Model:	BD Influx System (USB)
ValComp:	7.5.1.3.16	Cytometer Serial #:	X64650000106

Details

Data Source:	Cytometer	Sort Mode:	1.0 Drop Pure
Nozzle Diameter (µm):	0.00	Drop Envelope:	1.0 Drop
Sheath Pressure (PSI):	0.00	Sort Objective:	Purify
Sort Device:	4 Tube Holder - 4 Way Sort	Phase Mask:	16/16
Piezo Amplitude:	7.40	Extra Coincidence Bits:	4
Drop Delay:	31.9000	Drop Frequency (kHz):	36.90

Sort Details

Name	Population	Event Limit	Event Count	Sort Count	Sort Rate	Aborts	Abort Rate	Efficiency	Time (sec)
Far Left	-	-	-	-	-	-	-	-	-
Left	P2	120,000	577,487	120,000	1,089	11,435	103	91.3%	110
Right	-	-	-	-	-	-	-	-	-
Far Right	-	-	-	-	-	-	-	-	-

Sample 3 _Rep1 :

Sort Report

System

Sort Start:	12/1/2021 2:01:58 PM	Server:	Utopex
Application:	BD FACS™ Sortware	Build:	1.2.0.108
Version:	1.2.0.142	Cytometer Model:	BD Influx System (USB)
ValComp:	7.5.1.3.16	Cytometer Serial #:	X5000032

Details

Data Source:	Cytometer	Sort Mode:	1.0 Drop Pure
Nozzle Diameter (µm):	0.00	Drop Envelope:	1.0 Drop
Sheath Pressure (PSI):	0.00	Sort Objective:	Purify
Sort Device:	4 Tube Holder - 4 Way Sort	Phase Mask:	16/16
Piezo Amplitude:	6.09	Extra Coincidence Bits:	4
Drop Delay:	37.8125	Drop Frequency (kHz):	36.80

Sort Details

Name	Population	Event Limit	Event Count	Sort Count	Sort Rate	Aborts	Abort Rate	Efficiency	Time (sec)
Far Left	-	-	-	-	-	-	-	-	-
Left	P3	Unlimited	264,742	150,983	817	2,383	12	98.4%	184
Right	-	-	-	-	-	-	-	-	-
Far Right	-	-	-	-	-	-	-	-	-

Sample 3 _Rep2 :

Sort Report

System

Sort Start:	12/16/2021 2:07:59 PM	Server:	Utopex
Application:	BD FACS™ Sortware	Build:	1.2.0.108
Version:	1.2.0.142	Cytometer Model:	BD Influx System (USB)
ValComp:	7.5.1.3.16	Cytometer Serial #:	X64650000106

Details

Data Source:	Cytometer	Sort Mode:	1.0 Drop Pure
Nozzle Diameter (µm):	0.00	Drop Envelope:	1.0 Drop
Sheath Pressure (PSI):	0.00	Sort Objective:	Purify
Sort Device:	4 Tube Holder - 4 Way Sort	Phase Mask:	16/16
Piezo Amplitude:	7.70	Extra Coincidence Bits:	4
Drop Delay:	31.9000	Drop Frequency (kHz):	36.90

Sort Details

Name	Population	Event Limit	Event Count	Sort Count	Sort Rate	Aborts	Abort Rate	Efficiency	Time (sec)
Far Left	-	-	-	-	-	-	-	-	-
Left	P2	120,000	297,686	120,000	1,301	4,729	51	96.2%	92
Right	-	-	-	-	-	-	-	-	-
Far Right	-	-	-	-	-	-	-	-	-

Sample 4 _Rep1:

Sort Report

System

Sort Start:	12/1/2021 1:56:52 PM	Server:	Utopex
Application:	BD FACS™ Sortware	Build:	1.2.0.108
Version:	1.2.0.142	Cytometer Model:	BD Influx System (USB)
ValComp:	7.5.1.3.16	Cytometer Serial #:	X5000032

Details

Data Source:	Cytometer	Sort Mode:	1.0 Drop Pure
Nozzle Diameter (µm):	0.00	Drop Envelope:	1.0 Drop
Sheath Pressure (PSI):	0.00	Sort Objective:	Purify
Sort Device:	4 Tube Holder - 4 Way Sort	Phase Mask:	16/16
Piezo Amplitude:	6.07	Extra Coincidence Bits:	4
Drop Delay:	37.8125	Drop Frequency (kHz):	36.80

Sort Details

Name	Population	Event Limit	Event Count	Sort Count	Sort Rate	Aborts	Abort Rate	Efficiency	Time (sec)
Far Left	-	-	-	-	-	-	-	-	-
Left	P3	Unlimited	313,569	151,099	716	3,410	16	97.8%	210
Right	-	-	-	-	-	-	-	-	-
Far Right	-	-	-	-	-	-	-	-	-

Sample 4 _Rep2:

Sort Report

System

Sort Start:	12/16/2021 2:04:52 PM	Server:	Utopex
Application:	BD FACS™ Sortware	Build:	1.2.0.108
Version:	1.2.0.142	Cytometer Model:	BD Influx System (USB)
ValComp:	7.5.1.3.16	Cytometer Serial #:	X64650000106

Details

Data Source:	Cytometer	Sort Mode:	1.0 Drop Pure
Nozzle Diameter (µm):	0.00	Drop Envelope:	1.0 Drop
Sheath Pressure (PSI):	0.00	Sort Objective:	Purify
Sort Device:	4 Tube Holder - 4 Way Sort	Phase Mask:	16/16
Piezo Amplitude:	7.82	Extra Coincidence Bits:	4
Drop Delay:	31.9000	Drop Frequency (kHz):	36.90

Sort Details

Name	Population	Event Limit	Event Count	Sort Count	Sort Rate	Aborts	Abort Rate	Efficiency	Time (sec)
Far Left	-	-	-	-	-	-	-	-	-
Left	P2	120,000	326,842	120,000	1,778	9,768	144	92.5%	67
Right	-	-	-	-	-	-	-	-	-
Far Right	-	-	-	-	-	-	-	-	-
