# Supplementary material for: p53 promotes revival stem cells in the regenerating intestine after severe radiation injury
Source: Nat Commun. 2024 Apr 8;15:3018. doi: 10.1038/s41467-024-47124-8 (PMC11001929; doi:10.1038/s41467-024-47124-8)

Supplementary Figures

Supplementary Figure 1. Identification and transcriptional characterization of intestinal epithelial cell clusters identified by single-cell RNA sequencing.

(**A**) Schematic illustrating the pipeline on how epithelial cells were subset from the rest of the stromal and immune cells in the single-cell RNA sequencing analysis.

(**B**) UMAP showing the epithelial cell clusters identified in the single-cell RNA sequencing analysis.

(**C**) Dotplot indicating the expression of lineage-specific gene markers (columns) across all epithelial cell clusters (rows).

(**D**) UMAPs illustrating the expression of the enterocyte gene markers *Krt20* and *Alpi* across the epithelial cell clusters (top). Heatmap represents the expression of the villus zonation gene signatures across the enterocyte cell clusters (bottom).

(**E**) Heatmap representing the average expression of intestinal epithelial lineage gene signatures (Supplementary Data 2) across the epithelial cell clusters identified in the scRNA sequencing data. Dashed lines mark the label-retaining cell (LRC) gene signature.

(**F**) Identification of FCC and revSC clusters based on the expression of *Ly6a* and *Clu* genes.

(**G**) Violin plot showing the expression of *Lgr5*, *Pcna*, *Ly6a,* and *Clu* genes across the columnar crypt base (CCB1 and 2), transit amplifying (TA), fetal-like CBC cells (FCC), and revival stem cells (revSC) clusters.

(**H**) Percentage of cells in each intestinal epithelial cell cluster from non-irradiated intestines (d0) and two (d2) and three (d3) days after 12 Gy SBI.

(**I**) Representative sm-FISH images of *Clu* (left) or *Ly6a* (right) and *Mki67* mRNA molecules expressed in intestinal tissue sections from mice three days after irradiation. Scale bars=50μm.

(**J**) Quantification of double *Mki67+ Ly6a+* and *Mki67+Clu+* cells in intestinal tissue sections from mice 3 days after irradiation.

(**K**) Representative sm-FISH images of *Clu* and *Ly6a* gene expression in intestinal tissue sections from non-irradiated mice or 48h, 60h and 72h after 12 Gy SBI. Scale bars=50μm.

Supplementary Figure 1

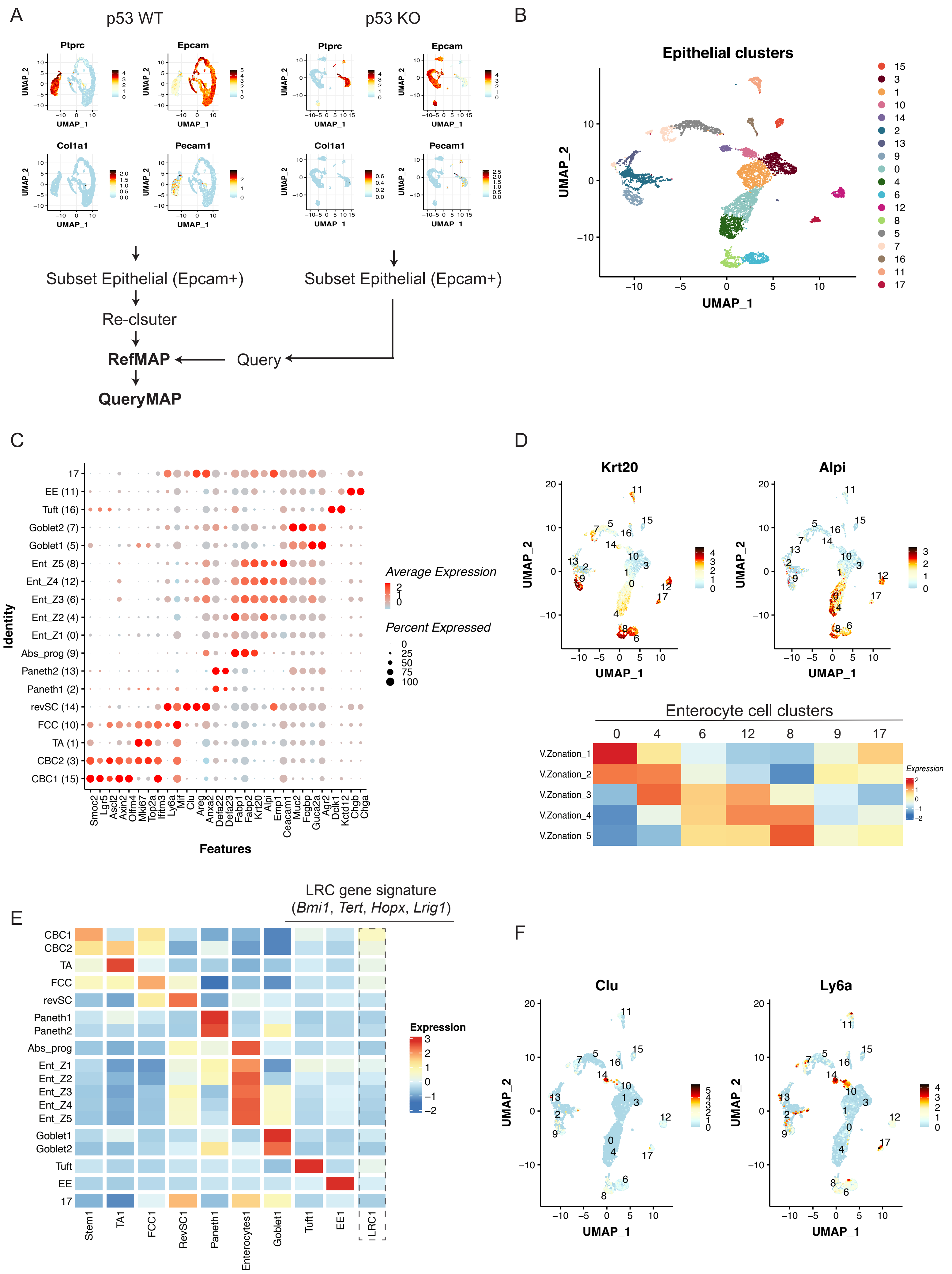

Supplementary Figure 1

G

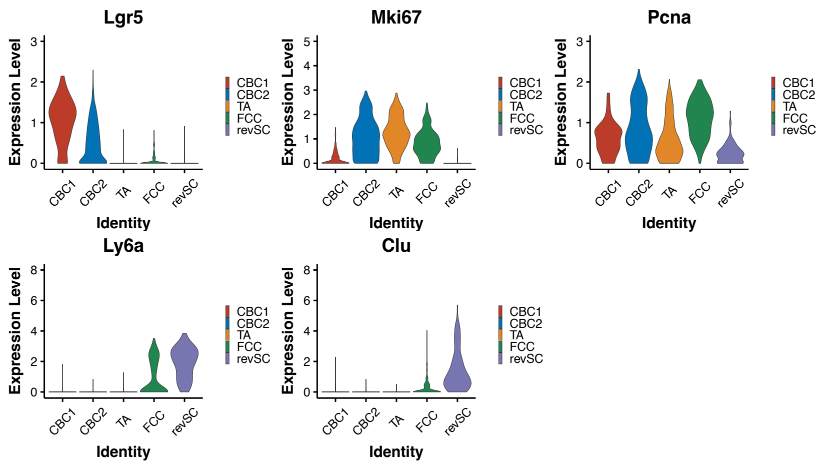

H

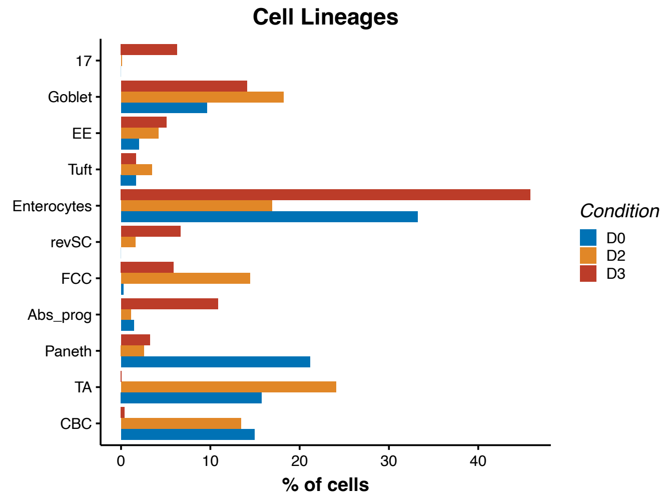

I

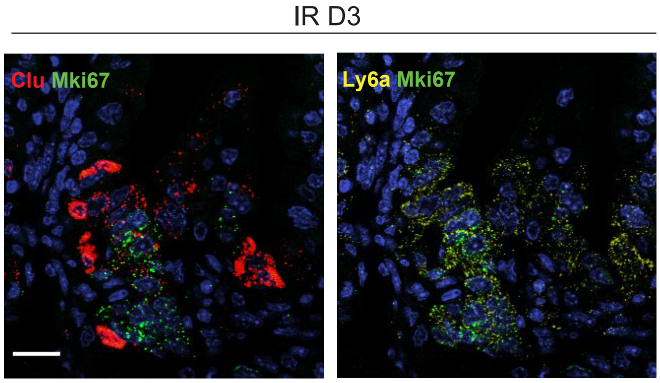

J

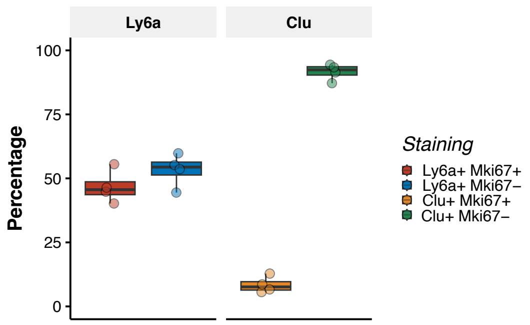

K

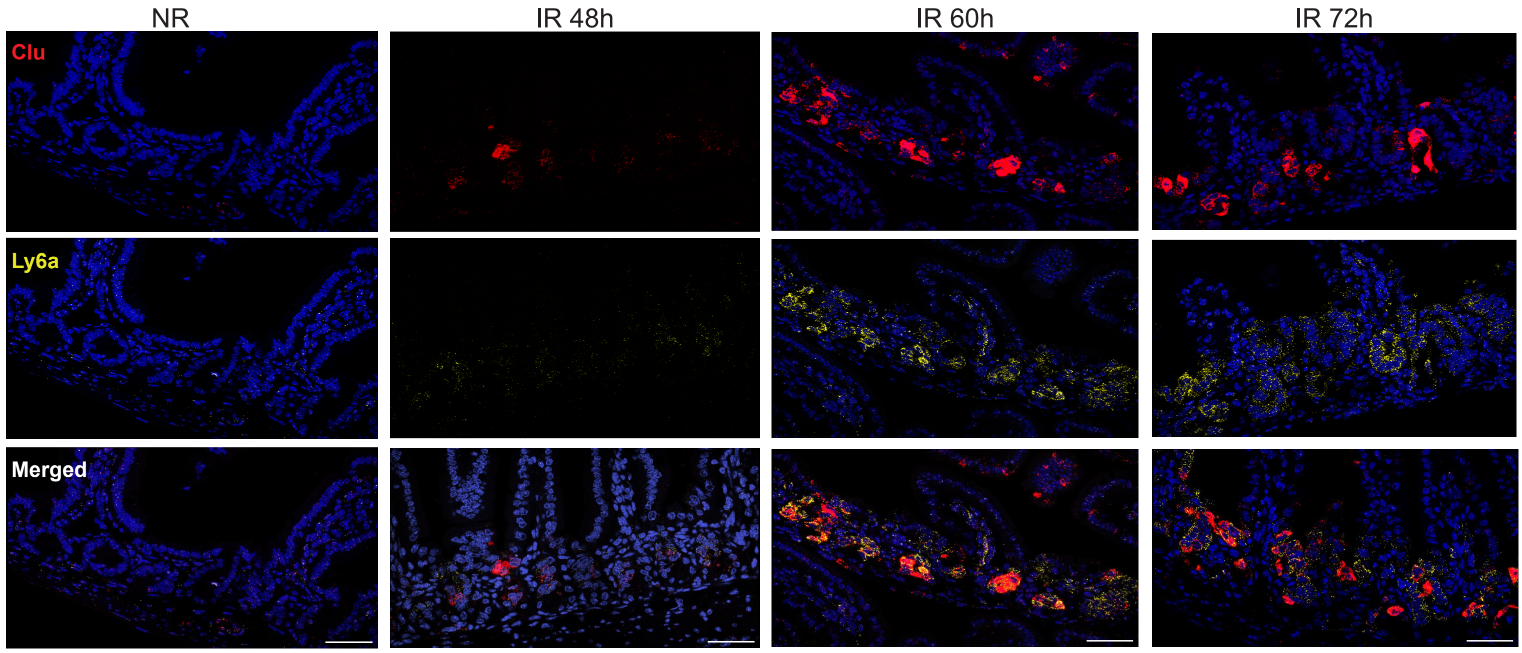

Supplementary Figure 2. Yap signaling is activated in revival stem cells (revSC) after intestinal irradiation.

(**A**) UMAP showing the transcriptional expression of downstream Yap target genes (Supplementary Data 2) across all intestinal epithelial cells (left) and in NR, day 2 and day 3 after irradiation (right).

(**B**) UMAP representation of single-cell transcriptome profile of intestinal epithelial cells from Lats1$^{fl/fl}$ Lats2$^{fl/fl}$ (WT) and Lrig-CreERT2- Lats1$^{fl/fl}$ Lats2$^{fl/fl}$ (Lats 1/2cKO) 7 days after tamoxifen induction. (Data obtained from *Cheung et al., Cell Stem Cell 2020*).

(**C**) Expression of revSCs associated genes (*Clu*, *Ly6a*, *Anxa1*, *Cdkn1a*) and p53 target genes (*Trtp53*, *Phlda3*, *Bax*, *Atg9b*) in WT or Lats 1/2cKO intestinal epithelial cells.

Supplementary Figure 2

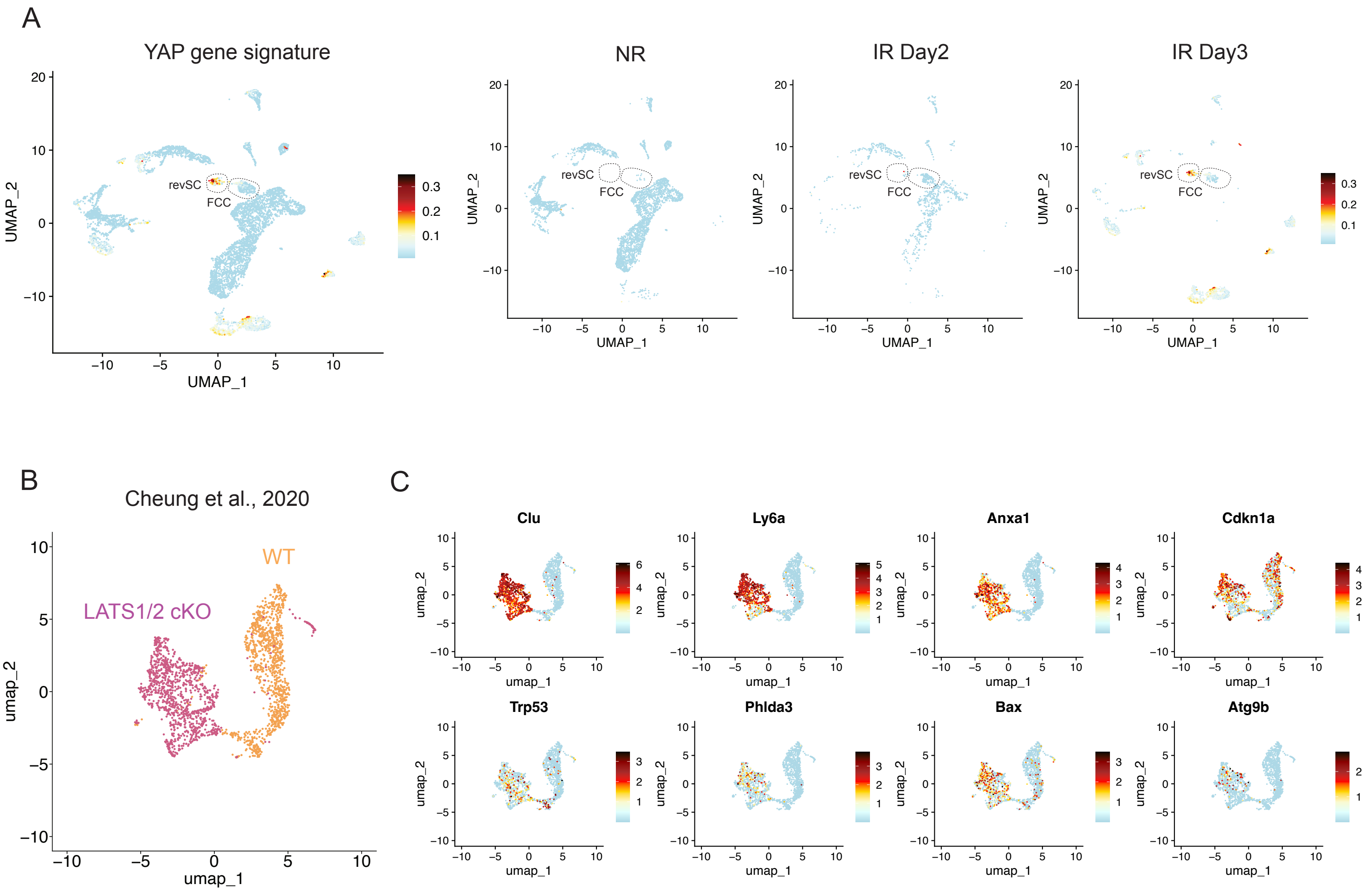

Supplementary Figure 3. Clu+ cells contribute to the regeneration of the small intestines following various doses of sub-total body irradiation.

(**A**) Schematic representing the lineage tracing model from Clu+ cells after IR damage in intestinal organoids in vitro. (Created with BioRender.com).

(**B**) Individual immunofluorescence channels (DAPI-blue, tdTOMATO-yellow, and Lgr-GFP-green) from Figure 3f.

(**C**) $\textit{Clu}^{\textit{CreERT2}}$; R26$^{\textit{LSL-tdTomato}}$ mice exposed to 0, 10 and 14 Gy SBI were injected with 100 mg/kg tamoxifen at 24 and 48 hours after irradiation to induce CreER-mediated gene recombination and expression of tdTomato. The small intestines were harvested from mice that survived 10 days after irradiation. (Created with BioRender.com).

(**D**) Kaplan-Meier curves of mice following 0, 10, or 14 Gy SBI (total n=16). P value is calculated using a long-rank test.

(**E**) Representative tissue sections of the small intestine harvested 10 days after 0, 10 and 14 Gy SBI. The DAPI signal is shown in blue and the tdTomato signal is shown in red. Scale bars=500µm.

(**F**) Quantification of lineage tracing events of the small intestines harvested 10 days after irradiation. The boxplot represents the interquartile range (IQR) of the data, with the median indicated. The whiskers represent the highest and lowest values within 1.5 times the IQR. Each dot represents one mouse (n=3/condition). Statistical significance was calculated using one-way ANOVA test followed by a Post-hoc Tukey's HSD test.

Supplementary Figure 3

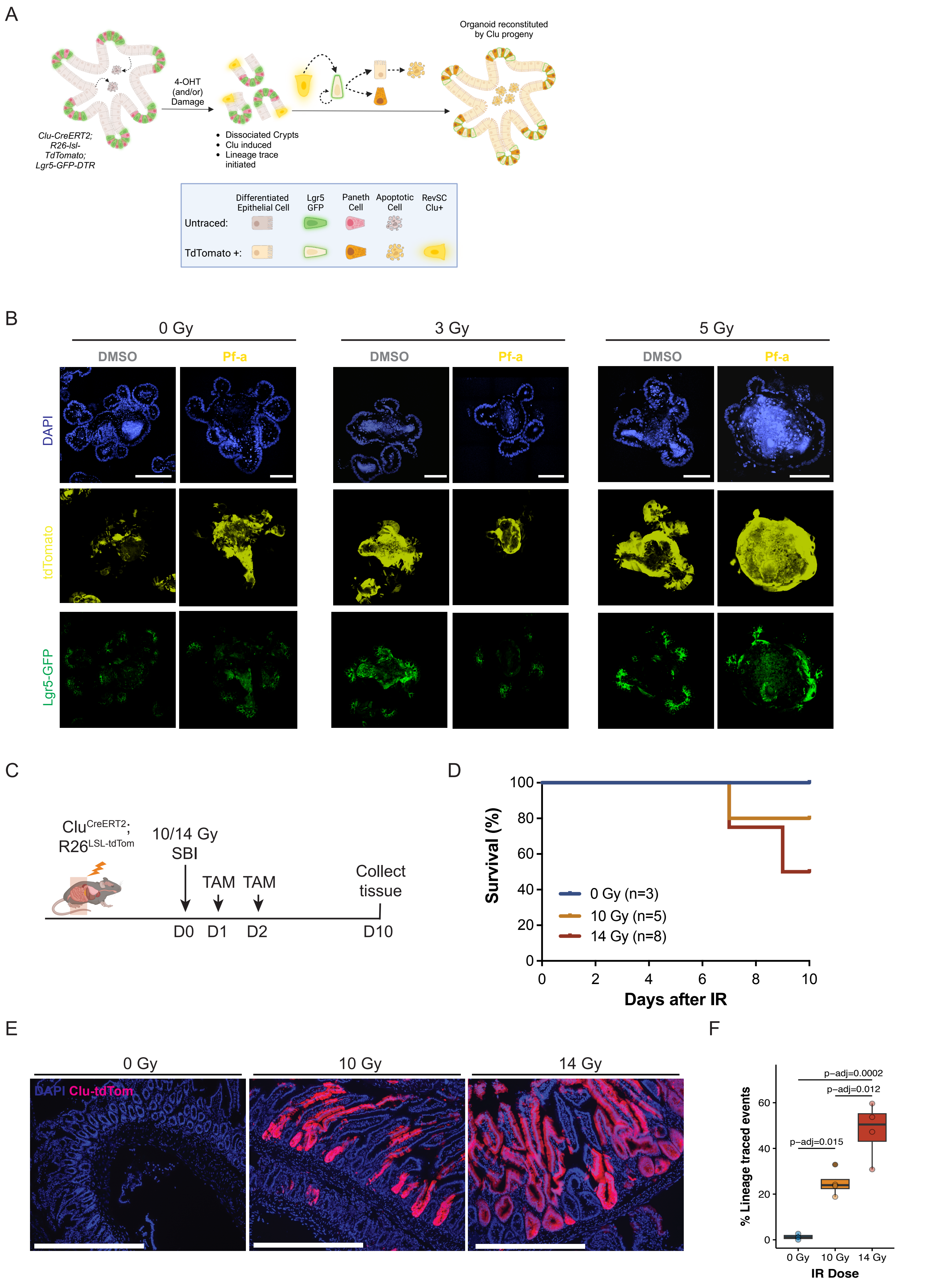

Supplementary Figure 4. Transient activation of p53 in intestinal epithelial cells after irradiation.

(**A**) Representative IHC staining of P53 protein in *Villin*^{Cre}*; p53*$^{FL/+}$ mice at various time points post-irradiation. The adjacent panel photo highlights an example of a positively staining crypt for each timepoint. Scale bars =100μm.

(**B**) Quantification of p53-positive cells per crypt in intestinal tissue sections from *Villin*^{Cre}*; P53*$^{FL/+}$ mice at various time points after irradiation. Data is presented as the average number of p53-positive cells per crypt quantified from 3 to 4 mice, with 4 different tissue areas assessed for each mouse.

(**C**) UMAP plots showing the *Mdm2* mRNA expression in revSC and FCC populations at day 0, or 2 and 3 days after 12 Gy SBI.

(**D**) Quantification of the percent of cells in organoids with nuclear p53 signal following irradiation and given 5 day regeneration period +/- Nutlin-3. Individual dots represent single organoids. Error bars represent the error of the mean. Statistical significance was calculated using a two-tailed t-test.

(**E**) Graphic representation of the working model. (Created with BioRender.com).

Supplementary Figure 4

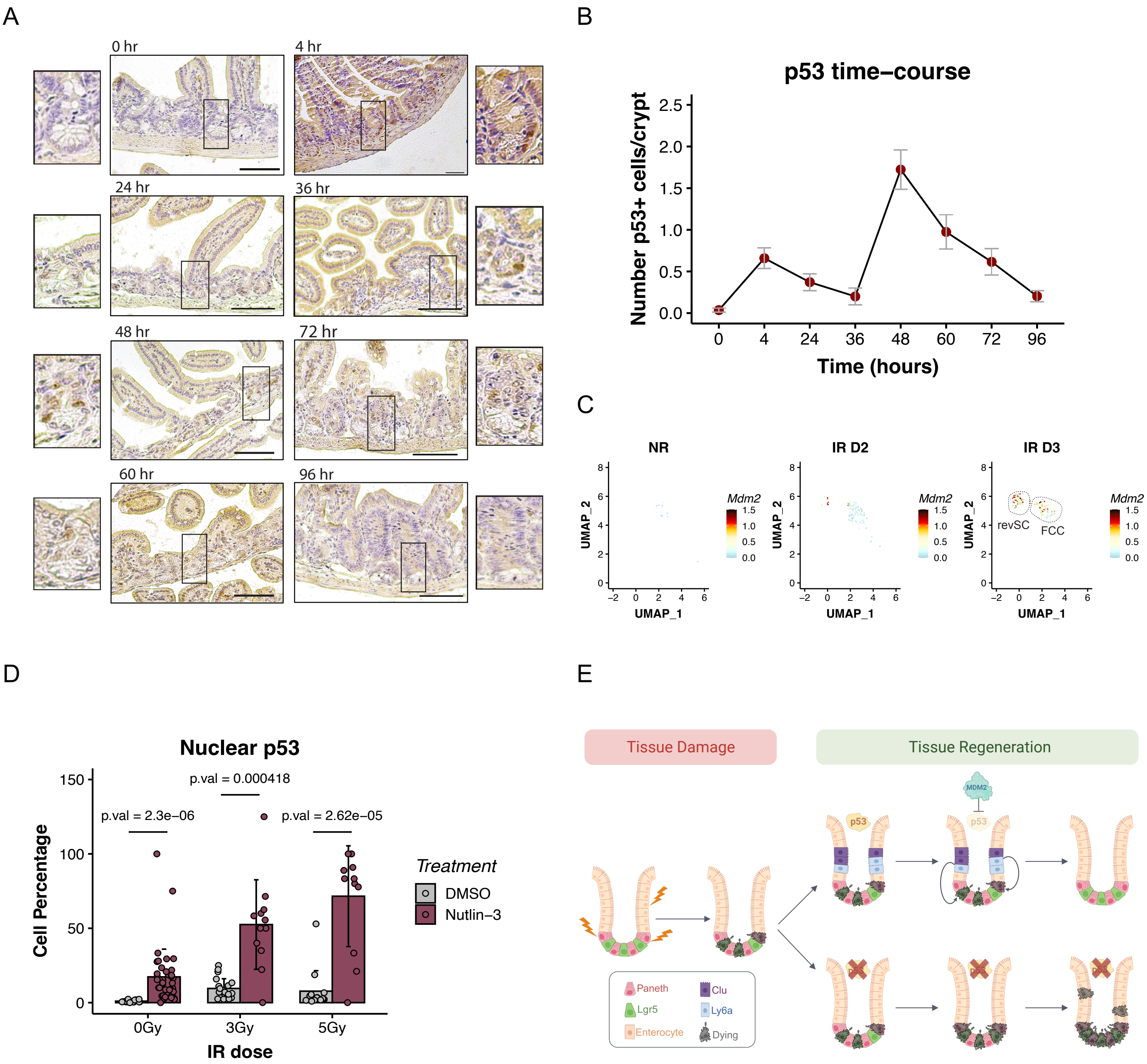

Supplementary Figure 5. P53 KO mice lack the expression of P53-downstream targets after IR in intestinal epithelial cells and are more sensitive to radiation-induced intestinal toxicity.

(A) Heatmap showing the expression of p53 transcriptional target genes in p53 WT (*Villin^{Cre}; p53$^{FL/+}$*) or p53 mutants (*Villin^{Cre}; p53 $^{FL/-}$*) in non-irradiated (NR) or two days after 12 Gy SBI.

(**B**) p53 IHC in the intestinal epithelium of p53 WT (*Villin^{Cre}; p53$^{FL/+}$)* or p53 mutants (*Villin^{Cre}; p53$^{FL/FL}$*) and p53 TAD mutants non-irradiated (No IR) or 4h after 12Gy SBI. Black arrows indicate nuclear p53 in epithelial cells. Scale bars =100μm.

(**C**) Representative images of immunohistochemistry staining of cleaved-caspase 3 (CC3) protein on intestinal tissue sections from p53 WT or p53 mutants. Images are from tissues collected from non-irradiated (No IR) mice or mice 4 hours after 13.3 Gy SBI. Scale bar=100um.

(**D**) Quantification of CC3-positive cells per crypt across genotypes and conditions. Data is presented as the average number of CC3-positive cells per crypt. The boxplot represents the interquartile range (IQR) of the data, with the median indicated. The whiskers represent the highest and lowest values within 1.5 times the IQR Each dot represents a different mouse (n=3-10 mice/genotype/condition). For each mouse, between 3-4 images from 4 tissue areas were quantified. Statistical significance was assessed using a two-sided t-test.

Supplementary Figure 5

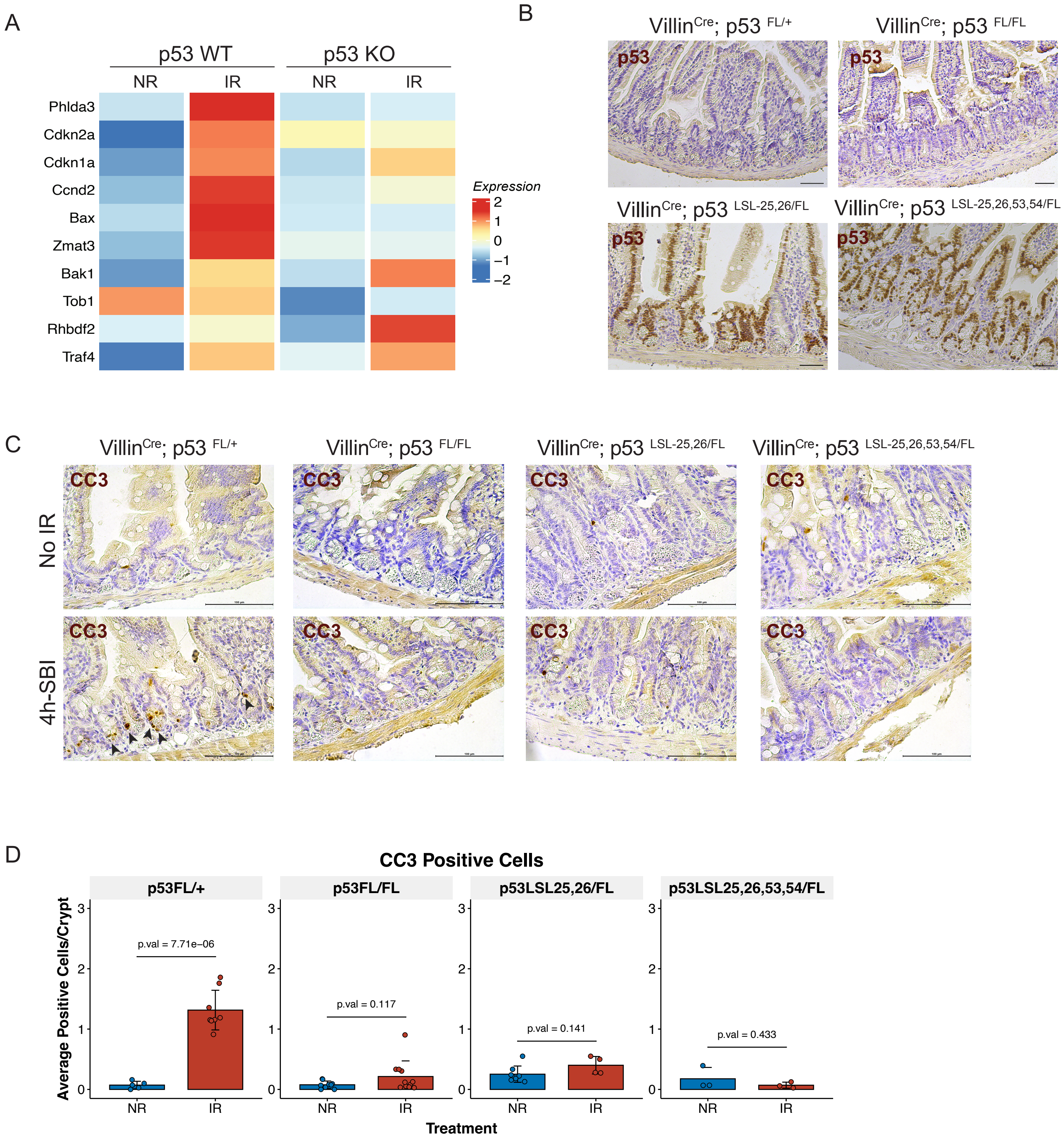

Supplement: Supplementary file 1 — Supplementary Information [file 41467_2024_47124_MOESM1_ESM.pdf]
